# Gender-dependent association between sleep duration and overweight incidence in CHINESE school children: a national follow-up study

**DOI:** 10.1186/s12889-018-5470-1

**Published:** 2018-05-11

**Authors:** Muqing Cao, Yanna Zhu, Xiuhong Li, Yajun Chen, Jun Ma, Jin Jing

**Affiliations:** 1 Department of Maternal and Child Health, Faculty of Public Health, Sun Yat-sen University, No. 74, Zhongshan 2nd Road, Yuexiu, 510080 Guangzhou, People’s Republic of China; 2 Institute of Child and Adolescent Health, School of Public Health, Peking University Health Sciences Center, No. 38, Xueyuan Road, Haidian, 100191 Beijing, People’s Republic of China

**Keywords:** Overweight, Sleep duration, Gender, Age, Adolescents, Longitudinal study

## Abstract

**Background:**

The relationship between sleep duration and overweight risk remains unexplored among Chinese children. This study aims to evaluate this association in a national investigation with school-aged population.

**Methods:**

There were 18,302 normal weight children in this Chinese national study which conducted during 2013–2014 included in the research. Anthropometric measurements were performed both at baseline and after 6–9 month. Sleep duration, physical activity, food intake and social economic information were collected by self-report questionnaire. Overweight was defined according to the updated Chinese criterion. Cox regression was used to evaluate the relationships between sleep duration and overweight incidence with multivariable adjusted.

**Results:**

In total, there were 443 new overweight cases recorded at the end of observation. Overweight incidence with greater than 9 h (long sleep duration, LSD), 7 to 9 h (middle sleep duration, MSD), and less than 7 h of sleep (short sleep duration, SSD) were 2.7, 3.1 and 3.3% respectively. Stratified by gender and compared with LSD, the hazard ratio (HR) of overweight for females with MSD was 1.60 (95% CI: 1.02–2.52). Stratified by age and gender, the HR in the group of MSD was 2.13 (1.20–3.77) in female aged 6–10 years and 0.24 (0.06–0.93) in female aged 15–17 years**.**

**Conclusion:**

The association between short sleep duration and overweight is age- and gender dependent. In group of small age and elder age, girls’ adiposity states are independently associated with sleep duration. Sleep recommendation is a potential preventive action for overweight/obesity among girls.

## Background

Sleep duration has decreased in the past few decades along with global modernisation [1]. Teenagers in the United States reportedly sleep for an average of less than 9 h in 2005 [2]. With rapid economic development and lifestyle changes, it is reasonable to speculate that the situation in China may be more severe. Based on regional data from 2013, half of Chinese teenagers failed to obtain 7 h of sleep per night, and 60% of Chinese primary school children sleep for less than 9 h [3]; however, no national data is available to describe China’s situation.

Short sleep duration is associated with metabolic alternation [4], which promotes energy reservation [5] resulting in weight gain. For instance, short sleep duration was observed to increase ghrelin and decrease leptin releases, in which the former stimulates appetite and the latter restrains it. Consequently, these people tend to increase food consumption. In this case, the association between sleep and childhood obesity has been widely studied, most of which have indicated a negative association [6–9]. To investigate causality, longitudinal study should be adopted; however, most current studies have used cross-sectional data [10–12]. Hence, taking this longitudinal study to facilitate an in-depth understanding of the association between sleep and adiposity state could benefit children at risk.

Apart from obesity, overweight could also be a risk factor for negative health consequence [13]; however, few studies have mentioned the effect of short sleep on overweight risk. A link between no-rapid eye movement sleep and overweight was suggested, but the research has a relatively small sample size [14]. Moreover, child overweight, as the intermediate state between normal weight and obesity, could be reversed, which means it is worthy of exploration as an independent negative outcome. In addition, studies have usually focused on adults rather than children with rapid growth rate [15]. The sleep and weight standard recommendations for children are different [16], and thus, their adiposity states and sleep should be explored separately.

Sleep duration and adiposity states are influenced by several factors. Energy balance related behaviours such as physical activity, sedentary lifestyle and food intake [3] play an important role, which increases the complexity of the causality test [17]. With the use of China’s regional data [3], we confirmed that short sleep duration could be an independent risk factor for childhood obesity, but a longitudinal research is still warranted to test causality.

In addition, adequate sleep duration decreases with ageing especially among children [3, 7]. Thus, we speculate that the same sleep duration may have varying impact on the adiposity states of children with different ages. Unfortunately, information on age-specific effect is quite limited. Moreover, the requirements for daily sleep duration may also vary due to the different hormone levels of boys and girls [17]. In this case, the effect of sleep duration on adiposity states could differ among boys and girls.

Although the relationship between sleep duration and adiposity states has been extensively investigated in children, data on age- and gender-specific effects have remained limited and inconclusive [3, 18]. Given this background, we aim to describe the sleep duration among school-aged Chinese children and explore whether short sleep duration is an independent risk factor for the overweight and its potential gender- and age-specific effects. In this study, we investigated (i) the distribution sleep duration across age and (ii) the association between short sleep duration and overweight incidence in 9 months among different age and gender groups. For possible confounding factors, we adjusted physical activity/sedentary behaviour, dietary intake and social economic states.

## Methods

### Participant enrolment

Seven areas in China (Beijing, Tianjing, Liaoning, Ningxia, Shanghai, Changsha and Guangzhou) were chosen as research centres, which are considered representative in population geography for north (Beijing, Tianjing), northeast (Liaoning), northwest (Ningxia), east (Shanghai), central (Changsha) and south (Guangzhou) part of China [19]. The recruitment of children and the survey of baseline information were described in the published protocol [19]. In this study, we enrolled 18,304 normal-weight children aged 6–17 years who agreed to participate in our national research and were assigned to the control group [19]. During the follow-up, a total of 4215 children still lacked information (missing data or unreached during the follow-up survey), and thus, the final sample was comprised 14,089 children. Compared with the remaining participants, those excluded from the study indicated no differences in age, percentage of males or baseline BMI. The study was approved by the Sun Yat-sen University Ethics Committee, and all parents/guardians of children signed the informed consent.

### Anthropometric measurement

Each child underwent an anthropometric measurement which included height (centimetre, cm) and weight (kilogram, kg) from September to November 2013. The detail of measurement procedure could be found in our previously published article [3]. The data on children that can be classified as overweight, obese, undernourished (Chinese criteria) [16] or suffering from obvious diseases or physical/mental deformities were excluded in the baseline analysis and follow-up.

### Questionnaire survey

A standardised questionnaire was designed to collect demographic data (examination date, birth date, gender, mother’s education level and monthly family income), puberty onset (whether a child has menstruation or spermatorrhea or not [20]), physical activity lifestyle (weekly hours of high- and middle-level physical activity, walking and sedentary) and dietary intake (daily intake of meat, sugar beverage, fruit and vegetable). The questionnaires were developed based on the information, motivation, and behavioural skills model [21] which was piloted and revised in the early stages of the project to be feasible for children/parents and teachers. The questionnaire was delivered to teachers in class during the week when anthropometric data were measured. Children were instructed to answer the questionnaire with their parents or guardians. Well-trained researchers ensured that the children would return the answered questionnaire to school. When all of the questionnaires were handed in, the researchers collected them from each class, and quality control was performed. Questionnaires that lacked five or more answers were returned to the children or their caregiver to have them answer the questions. In addition, there were 3% of the questionnaires rechecked within 1 week. We emphasised that the primary caregiver of the child should help answer the questionnaire considering their knowledge of the child.

The questionnaire items are described as follows:

The *monthly family income* was obtained by asking: “How much is your family’s monthly income?” The monthly family income was categorised as < 5000 and > 5000 yuan, as well as unwilling to answer.

*Mother’s education years* was derived by asking: “How many years did your mother spend in school?” Mother’s education years was categorised as ≤9, 10–12, 13–16 and ≥ 16.

*Puberty onset* was obtained by asking: “Do you already have menstruation (female)/emission (male)?” Puberty onset was categorised as either Yes or No.

*Sleep duration* was determined by asking: “How many hours each night do you spend on sleeping?” Sleep duration was categorised as < 7, 7–9 and ≥ 9 h [7].

#### Daily food intake

Fruit intake was obtained by asking: “How many servings of fruit do you usually eat each day? One serving of fruit is equivalent to the size of an adult fist. If you are unsure about the size of an adult fist, then please check the last page of the questionnaire.”

Vegetable intake was derived by asking: “How many servings of vegetables do you usually eat each day? One serving of vegetable is equivalent to the size of an adult’s fist. If you are unsure about the size of an adult fist, then please check the last page of the questionnaire.”

Sugar beverage consumption was determined by asking: “How many servings of sugar beverages do you usually take each day? One serving of sugar beverage is 250 millilitres.” Sugar beverage includes juice with sugar, soda (e.g. Coca-cola), milk drinks, energy drinks (e.g. Red Bull) and other beverages that contain sugar.

Meat intake was obtained by asking: “How many servings of meat do you usually eat each day? A serving of meat is equivalent to the size of an adult palm. If you are unsure about the size of adult palm, then please see the last page of the questionnaire.”

#### Daily physical activity and sedentary behaviour

High-level physical activity (HLPA) was obtained by asking: “How many hours each day do you usually do high-level physical activity? High-level physical activity refers to activities that cause people to experience extreme exhaustion, including basketball and football, as well as carrying a heavy load.”

Middle-level physical activity (MLPA) was determined by asking: “How many hours each day do you usually have middle-level physical activity? Middle-level physical activity means activities that cause people to mildly perspire and experience slight exhaustion, for example, bicycling, playing table tennis and badminton, but not walking.”

Walking was derived by asking: “How many hours each day do you usually walk? Walking includes those that occurred at school and home, commuting between school and home and merely for exercise.”

Sedentary behaviour was assessed by the question: “How many hours each week do you sit or lie down at school and home (excluding sleeping)?”

### Follow-up data collection and definitions of overweight

Follow-up data were collected from May to June 2014. The height and weight of the baseline normal-weight children were measured as described above. BMI was calculated by dividing weight in kilograms by height in meter squared (kg/m^2^). Overweight was defined according to the latest Chinese criteria [16].

### Statistical analysis

We used EpiData 3.0 software (EpiData Association, Odense, Denmark) for data input and Statistical Package for the Social Sciences (SPSS, version 22.0, IBM Corporation, New York) to analyse data. Descriptive statistics were calculated for continuous (presented as mean values and standard errors) and categorical variables (presented as proportions). Chi-square tests were carried out for the differences of categorical variables. For continuous variables, the general linear model was used to evaluate differences among sleep duration groups, and LSD tests were performed for post hoc comparison between groups. The risk of overweight was evaluated using Cox regression models initially adjusted for age and gender in Model 1, and then puberty onset, dietary intake, physical activity and sedentary behaviour, mother’s education and monthly family income were subsequently introduced into Model 2. The cluster data from the seven study areas were also introduced into the model, and a robust standard error was used to minimise the effect that the data may have obtained from different areas. The results were reported using hazard ratios (HRs) and corresponding 95% confidence intervals (CIs). *P* values of less than 0.05 were considered statistically significant. All the figures were prepared with Graph Pad 6.0 (La Jolla, CA, USA).

## Results

### Baseline characteristics of the study population

**Fig. 1:**
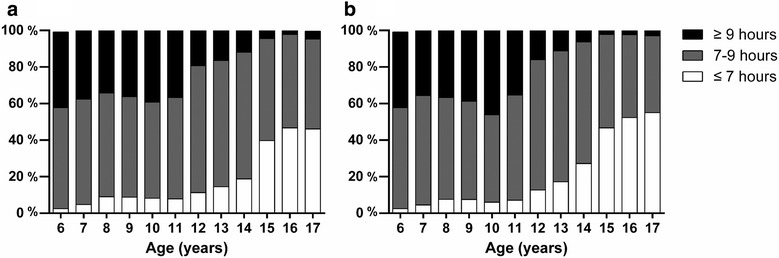
Distribution of sleep duration among normal-weight Chinese males (a) and females (b) aged 6–17 years

The baseline sample comprised of 18,302 normal-weight children (47% males). The baseline characteristics of the participants are described in Table 1. Compared with females, males indicated higher mean values in height, weight, BMI, daily intake of foods (fruit, meat and sugar beverages) and physical activity (all *p* < 0.001, Table 1), but lower mean value in sedentary behaviour (*p* < 0.001, Table 1). In total, 18.3% of the participants reported a sleep duration of less than 7 h (short sleep duration, SSD), whereas 24.4% reported over 9 h (long sleep duration, LSD) daily. More females reported SSD than males (*P* < 0.01; Table 1). The trend showed that Chinese normal-weight children tended to have less sleep hours as they grow up (Fig. 1).

**Table 1:** Baseline characteristics among children aged 6–17 years in urban Guangzhou, China

	Total	Boys	Girls	*P* value
Demographics				
No. of participants	18,302	8624	9678	–
Age (years)	11.32(3.16)	11.29(0.04)	11.49(0.04)	0.001
From Northern area (%)	40.4	40.5	40.2	< 0.001
Monthly income of Family (%)				0.012
≤ 5000 yuan	30.5	29.8	31.0	
> 5000 yuan	13.9	13.5	14.4	
Not willing to answer	55.6	56.7	54.6	
Mother’s education year (%)				0.01
≤ 9	43.7	55.0	51.9	
10–12	20.4	25.6	25.6	
13–16	11.9	11.0	12.6	
≥ 16	9.8	9.8	9.8	
Puberty on-set (%)	56.1	43.4	65.2	< 0.001
Height(cm)	147.26(16.12)	148.95(0.22)	146.93(0.21)	< 0.001
Weight(kg)	39.46(12.57)	40.56(0.17)	39.29(0.16)	< 0.001
BMI	17.73(2.26)	17.65(0.03)	17.73(0.03)	0.078
Dietary intake (servings/day)				
Fruit	1.27(1.06)	1.25(0.01)	1.30(0.01)	0.006
Vegetable	1.79(1.40)	1.81(0.02)	1.79(0.02)	0.27
Meat	1.17(1.21)	1.32(0.02)	1.02(0.02)	< 0.001
Sugar beverage	0.41(0.74)	0.50(0.01)	0.33(0.01)	< 0.001
Physical Activity (PA)				
HLPA (hour/day)	0.47(0.69)	0.61(0.01)	0.45(0.01)	< 0.001
MLPA (hour/day)	0.48(0.72)	0.53(0.01)	0.44(0.01)	< 0.001
Walking (hour/day)	0.78(1.13)	0.84(0.02)	0.74(0.02)	< 0.001
Sedentary (hour/week)	39.38(25.72)	38.40(0.35)	40.05(0.02)	< 0.001
Sleep duration (%)				
< 7 h	18.3	16.8	19.6	< 0.001
7–9 h	57.3	57.8	56.9	
> 9 h	24.4	25.4	23.5	

Continuous variables are displayed as mean (standard error, for boys and girls) and mean (standard deviation, for total), categorical variables are displayed as proportion. *P* values are from T-tests (continuous variables) and chi-square tests (categorical variables) between boys and girls

*BMI* body mass index, *PA* physical activity, *HLPA* high level physical activity, *MLPA* middle level activity

### Sample characteristic across sleep duration groups

The median follow-up time was 225 days. A total of 4215 participants were excluded due to lack of follow-up information (23.0%; no difference was found in age, gender, puberty onset, and distribution of sleep duration at baseline), whereas14,089 participants were included in the final analysis. The characteristics across the sleep duration of the participants, such as demography, dietary intake, physical activity/sedentary behaviour, anthropometry and incidence of overweight, are described in Table 2. Compared with children with LSD, participants with middle sleep duration (MSD, 7–9 h/night) and SSD indicated increased age, height, weight, BMI and puberty onset prevalence (all *p* < 0.05, Table 2). For physical activity/sedentary time, SSD and MSD children indicated decreased HPLA and MLPA but increased sedentary time compared with LSD children (all *p* < 0.05, Table 2). For dietary intake, SSD and MSD children reported increased meat and sugar beverage intake but decreased fruit and vegetable intake (all *p* < 0.05, Table 2). A total of 443 new overweight cases were recorded (3.1%), although no new obesity case was observed. Overweight incidents with LSD, MSD and SSD were 2.7, 3.1 and 3.3%, respectively.

**Table 2:** Baseline characteristics according to sleep duration among normal weight children aged 6–17 years

Characteristic	> 9 h	7–9 h	< 7 h	*P* for difference
Age group				
Before adolescents (male: 6–11 years, female:6–10 years)	2947	4293	551	< 0.001
Young adolescents (male: 12–15 years, Female: 11–14 years)	643	3044	823	
Elder adolescents (male: 15–17 years, Female: 16–17 years)	76	1285	1382	
Demographics				
No. of participants	3666	8622	2756	
Male (%)	48.0	46.4	42.2	< 0.001
Age (years)	9.64(0.06)	11.37(0.04)	13.53(0.07)	< 0.001
From Northern area (%)	36.8	37.2	39.7	< 0.001
Monthly income of Family (%)				< 0.001
≤ 5000 yuan	24.0	60.1	15.9	
> 5000 yuan	26.7	58.9	14.5	
Not willing to answer	23.9	55.0	21.0	
Mother’s education year (%)				< 0.001
≤ 9	24.4	59.1	16.5	
10–12	25.5	59.5	15.0	
13–16	27.6	58.9	13.4	
≥ 16	29.2	56.1	14.7	
Puberty on-set (%)	8.0	27.5	59.0	< 0.001
Lifestyle				
Physical activity(PA)				
High-level PA (hour/day)	0.56(0.14)	0.52(0.01)	0.52(0.02)	< 0.001
Middle-level PA (hour/day)	0.56(0.02)	0.47(0.01)	0.46(0.02)	< 0.001
Walking (hour/day)	0.80(0.02)	0.76(0.02)	0.83(0.03)	< 0.001
Sedentary (hour/week)	34.70(0.52)	40.03(0.33)	45.42(0.62)	< 0.001
Dietary intake(serve/day)				
Meat	1.14(0.02)	1.17(0.02)	1.25(0.03)	< 0.001
Sugar beverage	0.29(0.02)	0.41(0.01)	0.56(0.02)	< 0.001
Fruit	1.43(0.02)	1.26(0.01)	1.21(0.03)	< 0.001
Vegetable	1.94(0.03)	1.79(0.02)	1.71(0.03)	< 0.001
Anthropometry				
Height (cm)	139.77(0.31)	148.07(0.20)	156.82(0.363)	< 0.001
Weight (kg)	33.22(0.24)	39.93(0.15)	47.43(0.28)	< 0.001
BMI	16.65(0.05)	17.70(0.03)	18.86(0.05)	< 0.001

Continuous variables are displayed as mean (standard error), categorical variables are displayed as proportion

*P* values are from T-tests (continuous variables) and chi-square tests (categorical variables) between boys and girls

*BMI* body mass index, *PA* physical activity, *HLPA* high level physical activity, *MLPA* middle level activity

### Risks for overweight incidence among different sleep duration groups

Based on sleep duration, the HRs for the overweight participants are summarized in Table 3. After adjusting for all confounding factors (age, sex, physical activity/sedentary behaviour, dietary intake, mother’s education level and monthly family income), as stratified by gender, females in the MSD group indicated a 1.60-fold risk of overweight incidence (95% CI: 1.02–2.52) compared with those in the LSD group.

**Table 3:** HRs for overweight incidence according to sleep duration group among Chinese normal weight children in 6–9 months follow-up

Group	No. of Participants	No. of new overweight	Model 1^a^	Model 2^b^
Male				
> 9 h	1735	47	1	1
7–9 h	3950	112	1.09(0.77–1.54)	1.03(0.67–1.59)
< 7 h	855	38	1.38(0.87–2.21)	1.21(0.65–2.24)
Female				
> 9 h	1888	46	1	1
7–9 h	4563	140	1.23(0.87–1.74)	1.60(1.02–2.52)*
< 7 h	1667	50	1.28(0.81–2.00)	1.71(0.95–3.08)
Stratified by age and gender				
Before adolescents				
Male (6–11 years)				
> 9 h	1416	43	1	1
7–9 h	2148	66	1.04(0.71–1.53)	1.05(0.65–1.70)
< 7 h	302	7	0.84(0.38–1.86)	0.84(0.29–2.40)
Female (6–10 years)				
> 9 h	1499	34	1	1
7–9 h	2086	64	1.41(0.93–2.13)	2.13(1.20–3.77)*
< 7 h	247	8	1.46(0.68–3.16)	1.78(0.65–4.87)
Young adolescents				
Male (12–15 years)				
> 9 h	287	3	1	1
7–9 h	1411	34	2.14(0.65–7.01)	1.41(0.41–4.87)
< 7 h	450	17	3.58(1.02–12.49)*	2.19(0.56–8.51)
Female (11–14 years)				
> 9 h	346	7	1	1
7–9 h	1595	44	1.12(0.50–2.52)	1.31(0.49–3.47)
< 7 h	366	12	1.31(0.50–3.40)	1.28(0.38–4.28)
Elder adolescents				
Male (15–17 years)				
> 9 h	32	1	1	1
7–9 h	391	12	1.42(0.18–10.98)	0.36(0.04–3.46)
< 7 h	405	14	1.87(0.24–14.40)	0.28(0.03–2.89)
Female (16–17 years)				
> 9 h	43	5	1	1
7–9 h	882	32	0.50(0.19–1.29)	0.24(0.06–0.93)*
< 7 h	970	30	0.53(0.20–1.37)	0.28(0.07–1.12)

*HRs* hazard ratios, risk for overweight are displayed as HR (95%CI)

^*^P < 0.05 compared with ‘> 9 h’ group

^a^Model 1 was adjusted for age

^b^Model 2 was adjusted Model 1 covariates plus puberty on-set, food intake, physical activity& sedentary, domestic income and mother’s year of education

Based on the different puberty on-set age between female and male [22], we further stratified the sample by different age groups, females (aged 6–10 years) with MSD reported a 2.13-fold risk of overweight incidence (1.20–3.77; Table 3) compared with their counterparts in the LSD group. For elder adolescent (aged 15–17 years) females in the MSD group, the risk for overweight is 0.24 (0.06–0.93, Table 3) compared with those in the LSD group.

## Discussion

We revealed that insufficient sleep was fairly common among Chinese normal-weight children using a national follow-up study. At 6 years old, nearly 60% of boys and girls failed to obtain 9 h sleep per night, which was even worse among older ones. The association between sleep duration and overweight is dependent on children’s age and gender. For females, sleep duration between 7 and 9 h increases the risk of being overweight among those aged 6–10 year but decreases the risk for those aged 15–17 years (both compared with more than 9 h sleep). However, male’s sleep duration is not independently associated with the overweight incidence in each age group.

The decrease of sleep duration has recently become an international epidemic [23]. Based on the US recommendation, the optimal sleep duration for children should be over 9 h per night [24–26]. However, our study reported that 75% of children slept for less than 9 h (Table 1 and Fig. 1). This result was in accordance with China’s regional data that included normal weight and overweight/obesity children [3], but higher than the 44% reported by Spain in 2011 [27] and 11.1% reported by UK in 2015 [28]. On the one hand, the observed discrepancies may be explained by the academic burden which Chinese children generally have [29]; on the other hand, the population of our research included primary school children and adolescents, in which the latter delayed the sleep phase and retained an early school time [30]. As we observed, no previous studies have reported on the sleep time of normal weight children either in China or in other countries; however, these children usually report less sleep problems such as sleep apnea. Hence, we speculate that the prevalence of insufficient sleep could be slightly higher if we included children with overweight and obesity at the baseline.

Previous research from several other countries has also reported that short sleep duration is a risk factor for obesity and a high BMI-Z score [9], which is also mediated by other behaviour factors such as sedentary lifestyle [31], physical activity level and food intake [32]. However, the inner mechanism is still unclear, although most studies have tended to believe short sleep duration led to a combination of increased food intake and sedentary habits [33]. We observed less physical activity and more sedentary behaviour and sugar beverage intake in this study (Table 2), which supported the hypothesis mentioned above.

A few studies have explored sleep duration, as well as overweight and obesity from a gender-specific perspective [33, 34]. For instance, several studies have suggested that the effect of sleep duration on obesity was only present in females with considerable sleep debt [35]. In our study, increased risks of being overweight were observed only for females who slept for 7–9 h after multivariable adjustment (Table 2). In addition, the trend showed increased overweight risk for females with < 7 h sleep (although the statistic power compromised by a relative low sample size, as indicated in Table 3), which proved the gender specific phenomenon mentioned above [36]. Females reportedly need less sleep [36], and they may show different behaviour pattern for energy balance. Males have more physical activities and less sedentary behaviours (Table 1), which could evidently prevent them from being overweight; moreover, the higher basic metabolic rate of males compared with females increases energy expenditure [37], which could be a protective factor for overweight and obesity. In this case, the adiposity state may not be so sensitive to the short sleep decline among males relative to females. Furthermore, we also found reversed relationship between adiposity states and sleep duration among boys in our previous study [3], which also supports our results. In addition, the risk of overweight among females is observed when mother’s education and family income were adjusted (Table 3), which means that the social hierarchy of children depends on their sleep duration. This result is in line with those of previous studies [12]. A study in Japanese adults indicated that the association existed only in males [38]; however, it had limitation that the confounders in the study did not include behaviour. Although in our study a negative association among young adolescent males was observed (Table 2 and Model 1), but it tended to be insignificant after physical activity, food intake and social economic factor were included (Table 3 and Model 2). In this case, the association between sleep duration and overweight could be mediated by behaviour among males [38] rather than independent.

Stratified by age group, research has found that short sleep increased BMI [34] only in adolescent female. By contrast, we observed increased risk for overweight among before adolescence girls but decreased risk among elder adolescence girls with MSD (Table 3). The discrepancy among age groups may be attributed to the difference of age groups and the definition of short sleep first and second to declining biological sleep duration with age [39]. Although sleep recommendation for children is 9 h, it is well recognised that 6-year old children require more sleep than 17 years old [40],sleep duration declines naturally with age and puberty onset [30]. In addition, studies have indicated that long and short sleep duration increased the risk of overweight and obesity [7]. Moreover, it is reported that female sleep less than males [41]. Thus, the optimal sleeping hours for post-puberty females, which is less than 9 h, is possible, and 7–9 h sleep could be suitable for elder adolescent girls.

This study included some limitations. Sleep duration was self-reported, which sometimes could be the ideal sleep duration that the participants thought they had other than their actual sleep duration. In addition, nearly 23% of the participants failed to complete the follow-up procedure. Considering that overweight children may refuse to be weighed, the excluded subjects may have a higher rate of obesity. Bedtimes have been found to be associated with obesity independent of sleep duration [42]; however, we did not include this factor in the study. An objective technique is considerably useful in conducting sleep research in the future. The exclusion of self-report bias is generally deemed to be the optimal approach. Considering that we used a questionnaire that has neither been statistically validated nor tested for reliability; thus, potential bias cannot be excluded.

## Conclusion

In this study, we showed that sleep duration among normal-weight Chinese children decreased with age. For 6–10-year-old females, 7–9 h of sleep increased the risk of overweight relative to more than 9 h of sleep. However, the same sleep duration may decrease the risk for females aged 15–17 years, and males may not be as susceptible as their counterparts to the effect of sleep duration change in the adiposity state. For the purpose of obesity intervention, an age- and gender-specific sleep duration recommendation for children is warranted.

## Abbreviations

BMI: Body mass index
LSD: Long sleep duration
MSD: Middle sleep duration
SSD: Short sleep duration
